# Evaluation of hard and soft tissue changes around implant in partially edentulous patients: a clinico-radiographic study

**DOI:** 10.11604/pamj.2021.38.378.27870

**Published:** 2021-04-19

**Authors:** Nidhi Mehrotra, Amrinder Singh Tuli, Megha Phogat Rana, Rohit Singh, Avnish Singh, Vivek Singh

**Affiliations:** 1 Department of Periodontics, Seema Dental College and Hospital, Rishikesh, Uttarakhand, India,; 2 Department of Pedodontics and Preventive Dentistry, Vananchal Dental College and Hospital, Garhwa, Jharkhand, India,; 3 Department of Public Health Dentistry, Seema Dental College and Hospital, Rishikesh, Uttarakhand, India

**Keywords:** Implants, peri-implant soft tissue, crestal bone loss

## Abstract

**Introduction:**

implant supported prosthesis has become a viable treatment option for missing teeth. An important tool to detect early changes around implants is the standardized assessment of peri-implant hard and soft tissue parameters. The purpose of this prospective study was to clinically and radiographically assess the soft and hard tissues around implants.

**Methods:**

ten (10) patients with 13 implant supported prosthesis were included in the study. Clinical parameters plaque index (PI), gingival index (GI), modified sulcus bleeding index (mSBI), peri-implant Probing Depth (PD), gingival margin Level, width of keratinized mucosa (WKM) and implant mobility were measured at loading and at 3 and 6 months. The radiographic crestal bone loss and peri-apical implant radiolucencies were also evaluated at loading and at 3 and 6 months. Student paired t test and correlation and regression analysis was done to evaluate the effect of clinical variables over bone loss.

**Results:**

there was decrease in the site specific PI, GI, mSBI and peri-implant PD and an increase in the gingival recession from baseline to 6 months. The WKM remained stable throughout the study. Significant crestal bone loss was observed around implants more on the distal as compared to the mesial aspect. No mobility or peri-apical implant radiolucency was observed. Regression analysis of the confounding variables with bone loss showed no significant effect.

**Conclusion:**

the occlusal loading of implants after 6 months showed significant bone loss (<1mm), which was within acceptable limits and the soft tissues around implants were in good health.

## Introduction

Teeth are one of the major components of stomatognathic system, which provides a pleasing smile in addition to its functional aspect of mastication. Loss of tooth/ teeth results in loss of structural balance, inefficient oral function, poor esthetics and positional change of remaining natural teeth [1]. The clinical replacement of lost natural teeth by osseointegrated implants has become one of the major resolutions in prosthetic dentistry that provides comfort as well as patient´s satisfaction [1,2]. Osseointegration, being the main stay in implant dentistry, has been the ultimate goal for the dentists to achieve, and one of the pre-requisites for this to happen is that the immediate milieu around the dental implant must be conducive for proper healing and tissue regeneration [3]. The conditions of the soft and the hard tissues around dental implants play an important role in its success [1]. The peri-implant tissue is an adaptation of the masticatory mucosa. It is composed of connective tissue coated by layers of epithelial cells that attach to the implant surface forming the junctional epithelium [4]. The levels of supporting bone and surrounding soft tissue dimensions around single implants are essentially governed by the surgical and prosthetic parameters and their variables. The relationship of the position of the implant and its proposed restoration should be based on the implant shoulder, as this is presumed to influence the final hard and soft tissue response. Other factors, such as the presence of keratinized mucosa also play a significant role in the final position of the soft tissue around implants [5]. The initial stages of a peri-implant inflammation sometimes show only subtle signs of pathology progression. The state of peri-implant health is monitored clinically by visual inspection, measurement of a potential loss of hard tissue attachment, and the determination of inflammatory signs in terms of plaque accumulation (frequently causing the inflammation) and soft tissue bleeding on probing (BOP) and suppuration (reflecting the state of inflammation) [6].

Several periodontal diagnostic parameters have been proposed as markers of health or disease. Plaque and bleeding indices may be used to evaluate oral hygiene and muscosal inflammation. Probing depth and mobility are also frequently considered clinical parameters [7]. As the bone level constitutes the base for the supracrestal soft tissue, and evidence supports the existence of a 'biologic width' of the supracrestal soft tissue around the implant similar to that defined for the natural tooth, the level of the bone crest surrounding the implant is of utmost significance to determine the success of the implant. Bone loss may negatively influence the soft tissue topography and the esthetic outcome of the implant surgery [8]. Radiographic analysis has shown that the largest amount of bone loss occurs following implant placement and abutment connection [2]. Thus, continuous evaluation of patients treated with osseointegrated implants is necessary to determine the long-term success of the dental implant system used, to ascertain factors affecting the success of therapy and to identify method-specific problems [7]. The present study was undertaken to evaluate the changes in the hard and soft tissues around dental implants after prosthesis fabrication by assessing various clinical parameters such as Width of Keratinized Tissue (WKT), Gingival Margin Levels (GM), plaque index (PI), gingival index (GI), probing depth (PD) and implant mobility and also evaluating the implant radiographically for peri-apical implant radiolucencies and crestal bone loss after loading of implants.

## Methods

**Study subjects and location:** a total of 32 systemically healthy individuals, both male and female, within an age group of 18-65 years, having a single or multiple missing teeth replaced by implant supported prosthesis placed in the Seema Dental Implant Centre, Seema Dental College and Hospital, Rishikesh from January 2015 to March 2016 were evaluated for the study.

**The exclusion criteria for the study were as follows:** subjects with conditions requiring routine prophylactic use of antibiotics; subjects with endocrine disorders (example diabetes mellitus) or other systemic diseases; subjects with dental history of bruxism, or para-functional habits; smokers and subjects with adverse habits of pan, tobacco or betel nut chewing. After screening, 15 subjects with 25 loaded implants fulfilled the criteria and were included in the study. Five subjects with 12 implants were dropped out from the study after baseline as they did not report for the follow ups and finally a total of 10 subjects with 13 implants were reviewed further.

**Figure 1:**
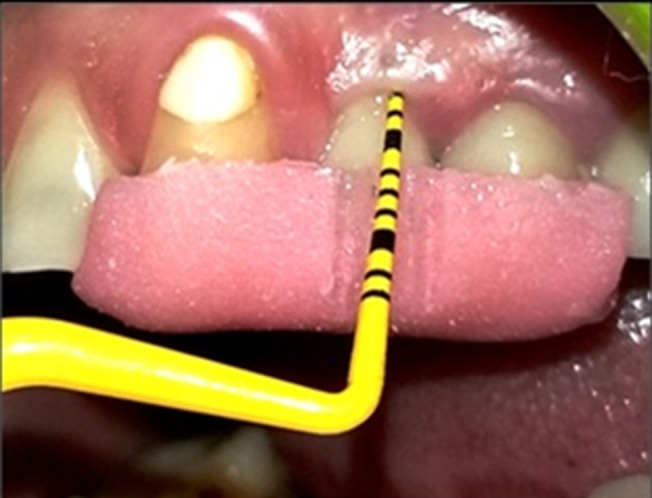
measurement of peri-implant probing depth

**Figure 2:**
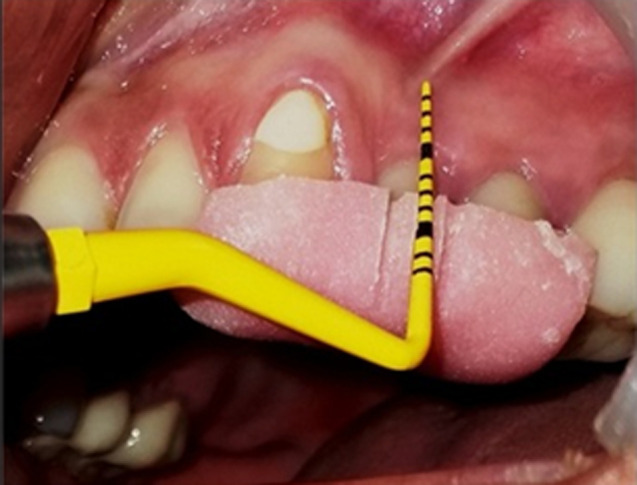
measurement of width of keratinized mucosa

**Figure 3:**
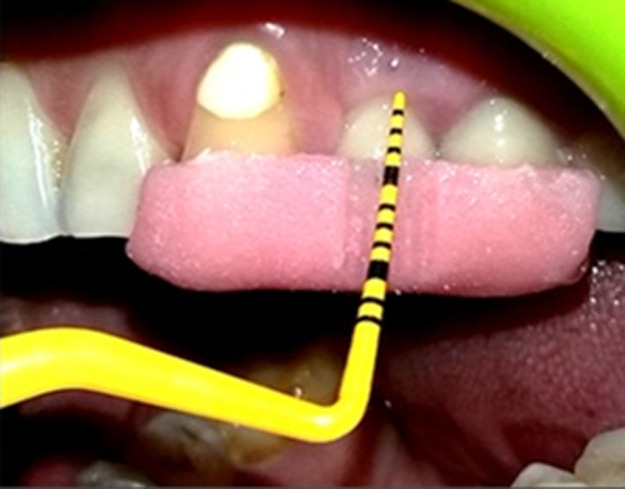
measurement of gingival margin level

**Figure 4:**
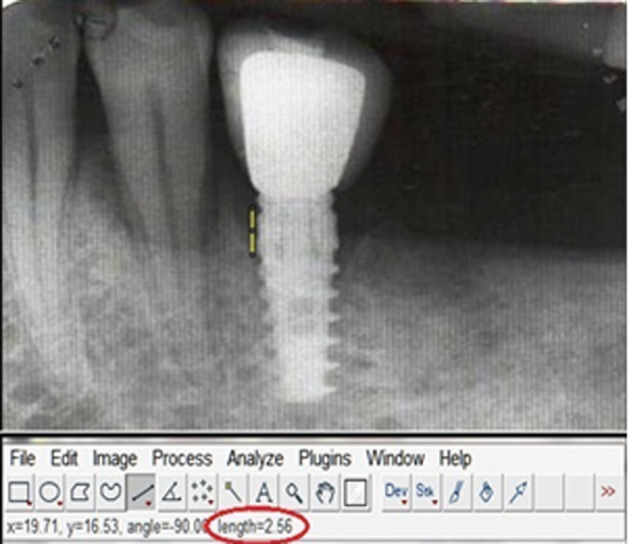
image J software showing measurement of distance from bone implant contact to implant abutment junction

**Standardization:** to standardize the reproducibility of clinical measurements, occlusal acrylic stents were fabricated with cold cure acrylic resin on a cast model of patients. The occlusal stents were made to cover the occlusal surface of the implant supported prosthesis and the occlusal surfaces of at least one tooth in the mesially and distally. Stents were extended apically on the buccal and lingual surfaces to cover the coronal third of the teeth. Grooves were made on the stent for reproducibility of probe position for measurements at the follow up visits. The following clinical parameters were recorded around each implant using a plastic UNC-12 periodontal probe: plaque index (PI), gingival index (GI), modified sulcus bleeding index (mSBI), probing depth (PD) (Figure 1), Width of keratinized tissue (WKT) (Figure 2) and Soft tissue level (Figure 3). Implant stability was also assessed manually using clinical implant mobility scale. Also, intra-oral periapical (I.O.P.A.) radiographs were taken using the long cone paralleling technique and they were scanned at 600 dpi. The bone levels were then measured as the distance from the fixed reference point on the implant, i.e., the implant-abutment junction to the first bone-implant contact (BIC) using Image J software as shown in (Figure 4) [3]. The presence of any peri-apical radiolucency around the implant was also assessed. All these parameters were recorded at baseline, i.e., at the time of prosthesis placement and at 3 and 6 months.

**Data analysis:** the data was compared using the Student´s Paired 't' test and the radiographic cone loss was correlated with the clinical variables using the Pearson´s correlation. Linear Regression Analysis was used for assessing the effect of independent variables over the dependent variable, i.e. bone loss.

## Results

The baseline, 3 and 6 months data from 13 implant sites was compared for changes in the clinical and radiographic parameters. The clinical parameters recorded are represented in (Table 1). The plaque index, gingival index and modified sulcus bleeding index decreased from baseline to 6 months and the differences were statistically significant. The mean peri-implant probing depth was 2.2 ± 0.6 at baseline, which increased to 2.35 ± 0.88 after 3 months, and then reduced to 2.25 ± 0.59 after 6 months of loading. The implant site also showed some amount of recession from baseline to 6 months. The differences in the peri-implant probing depth and soft tissue levels were however, statistically not significant. The mean keratinized mucosa index scores at the implant site recorded at baseline, 3 months and 6 months were same and all the implants showed 100% stability during the study. The radiographic analysis showed crestal bone loss around implants as shown in (Table 2); however the bone loss from baseline to 6 months was significant on the distal aspect as compared to the mesial aspect of the implants. No peri-apical implant radiolucency was observed around the implants at any point of time in the study.

**Table 1:** comparison of clinical parameters at the implant site at baseline, 3 and 6 months

Parameters	Time Interval	Mean ± SD	Difference from baseline	‘p' value
**Plaque index**	Baseline	1.45 ± 0.51	-	**-**
	3 months	0.9 ± 0.45	0.55	0.000
	6 months	0.75 ± 0.40	0.7	0.009
**Gingival index**	Baseline	1.7 ± 0.27	**-**	**-**
	3 months	1.4 ± 0.29	0.3	0.02
	6 months	1.35 ± 0.22	0.35	0.037
**Sulcus bleeding index**	Baseline	1.0 ± 0.71	**-**	**-**
	3 months	0.85 ± 0.74	0.15	0.07
	6 months	0.80 ± 0.51	0.2	0.242
**Peri-implant probing depth**	Baseline	2.2 ± 0.60	-	**-**
	3 months	2.35 ± 0.88	- 0.15	0.468
	6 months	2.25 ± 0.59	- 0.05	0.704
**Gingival margin level**	Baseline	5.0 ± 2.0	**-**	
	3 months	5.2 ± 2.28	- 0.2	0.16
	6 months	5.6 ± 1.67	- 0.4	0.374
**Width of keratinized mucosa**	Baseline	1.20 ± 1.10	**-**	**-**
	3 months	1.20 ± 1.10	-	-
	6 months	1.20 ± 1.10	-	-

**Table 2:** comparison of radiographic bone loss around implant at baseline, 3 and 6 months

Parameters	Time Interval	Mean ± SD	Difference from baseline	'p' value
**Mesial bone loss**	**Baseline**	1.48 ± 0.85	-	-
	3 months	1.70 ± 0.79	- 0.222	0.104
	6 months	2.17 ± 0.93	- 0.696	0.085
**Distal bone loss**	**Baseline**	1.92 ± 0.93	-	-
	3 months	2.22 ± 0.80	- 0.3	0.033
	6 months	2.47 ± 0.85	- 0.55	0.006

**Correlation of clinical parameters with bone loss:** (Table 3) depicts the correlation of clinical parameters with bone loss. Pearson´s correlation at baseline showed that the mesial and distal bone loss correlated with each other significantly with p value of 0.039. Also the gingival index score at the implant site correlated with the distal bone loss significantly with a p value of 0.035. Pearson´s correlation for percentage changes in the variables with peri-implant bone loss; only peri-implant probing depth showed a statistically positive correlation of 0.695 with bone loss at distal aspect (p = 0.008). Correlation with all other variables had statistically non-significant results. Also, the linear regression analysis showed that no clinical variable had any significant effect on bone loss around implants at both mesial and distal aspects.

**Table 3:** correlation of mesial and distal bone levels with other variables at 6 months

		Bone level mesial	Bone level distal	Plaque index Site	Gingival index Site	Bleeding index Site	Probing depth	Keratinized Mucosa width	Gingival margin level
**Bone level mesial**	**Pearson Correlation**	1	.820**	-0.16	0.003	-0.041	-.638*	-0.119	-0.363
	**p value**	-	0.001	0.601	0.991	0.894	0.019	0.699	0.223
**Bone level distal**	**Pearson Correlation**	0.820**	1	-0.197	-0.095	-0.038	-0.515	0.059	-0.432
	**p value**	0.001	-	0.518	0.758	0.901	0.072	0.848	0.141

## Discussion

The challenge of replacing missing tooth without compromising the neighbouring dentition has long confronted the dental profession [9]. Many different oral implant systems have been developed and promoted for the treatment of partially or completely edentulous patients. Continual evaluation of patients treated with implants is necessary to determine the long-term success, to ascertain factors affecting the success of therapy and to identify method-specific problems. Also, there is a growing need for evaluating esthetics around implant supported prosthesis [10]. Many clinical signs of failure emerge only when an irreversible and incurable state has already been reached. Thus, the parameters used routinely during maintenance of patients treated with implants should be sensitive enough to allow discrimination of early changes. These parameters should be easy to measure and yield reliable and reproducible information. To establish a set of useful clinical parameters, questions emerging during treatment and the practical consequences of missing information should be considered [10]. Thus, the present study evaluated the dimensional alterations of the peri-implant hard and soft tissues after loading the implants using various clinical and radiographic parameters. The establishment and maintenance of intimate contact between bone and implant is a major requirement for implant success. Absence of mobility is, therefore, an important criterion for assessing implant success. Clinically visible mobility of an implant after an appropriate healing period indicates failure to achieve osseointegration. All the implants evaluated in this study did not show any amount of mobility [10].

Plaque is considered an important etiological factor in peri-implantitis. It is therefore appropriate to monitor oral hygiene [10]. Lindquist *et al*. [11] found a significant relationship between oral hygiene and bone resorption over a 6-year period. Recognition of the signs of inflammation is important for early diagnosis of the peri-implant disease. In present study, there was a decrease in plaque scores, gingival index and sulcus bleeding index from baseline to 6 months which was statistically significant for both implant site and full mouth. The decrease were in accordance with the studies of de Angelo *et al*. [12] and Joly *et al*. [4] and can be attributed to the plaque control by the patient and the repeated reinforcements of the oral hygiene measures given to the patient by the clinician. The importance of keratinized mucosa (KM) surrounding the implant as a barrier against microorganisms and subgingival plaque as a factor for long-term success has been discussed [13]. Debate continues about whether it is necessary to have a zone of keratinized tissue surrounding implants [14]. keratinized mucosa is supposed to be a physical barrier, and the absence of it seems to provide easier apical migration of inflammation. A lack of keratinized gingival may create an environment that is less amenable to oral cleansing and more susceptible to irritation and discomfort during routine procedures. Dental implants with less than 2mm of KM seem to be more prone to recession and alveolar bone loss [13]. A study by Chung DM *et al*. has reported association between width of keratinized mucosa and gingival inflammation and plaque accumulation that is in accordance with the present study [15].

The results of the present study showed that the width of keratinized mucosa remained constant over time. However, 3 sites showed complete lack of keratinized mucosa at baseline, which was found to be associated with higher plaque accumulation and gingival inflammation as compared to the sites with keratinized mucosa. Warrer *et al*. [16] demonstrated that implants placed in areas lacking keratinized gingival had a higher susceptibility to tissue breakdown due to plaque accumulation. Also, the study group with < 2mm of keratinized mucosa around implants had significantly higher parameters (Plaque index, gingival index, bleeding on probing and marginal recession) in the study by Adibrad M *et al*. [17]. The present study showed an increase in the midfacial gingival margin levels indicating gingival recession, which was statistically not significant. The findings of this study are in accordance with the study by De Rouck *et al*. where the midfacial soft tissue showed little variation over time in the immediate as well as in the delayed loading groups but the change was not statistically significant [18]. Radiographically, a mean crestal bone loss of ≥1.5mm during the 1st year after loading and ≥0.2mm/year thereafter has been proposed as one of the major success criteria by Albrektsson [19]. The present study showed a significant increase in bone loss on both mesial and distal aspects of implant from baseline to 6 months, and the results are in accordance with the studies of De Rouck *et al*. Chou *et al*. and Kan *et al*. which showed a significant increase in the crestal bone loss after loading of implants [18,20,21]. The present study, however, showed more bone loss on the distal aspect as compared to the mesial, which is in contrast to the retrospective study by Fernandez *et al*. [22] which showed more bone loss on the mesial aspect of the implants over a 12 months period. The overall observations of the study showed crestal bone loss around implants which was well within the limits as described in literature. The subjects in the present study maintained an acceptable oral hygiene throughout the study period which was observed as decrease in the scores of plaque, gingival and bleeding indices. In accordance to the findings of the present study, Patil *et al*. Chung *et al*. and Adibrad *et al*. have also reported no significant association of peri-implant soft tissue health and width of keratinized mucosa with bone loss around implants [5,15,17]. Also, longitudinal studies by Romeo *et al*. and Cecchinato *et al*. have demonstrated that loss of alveolar bone may be absent or minimal in well maintained implants [23,24].

## Conclusion

The present study was conducted for a period of 6 months. All the implants showed <1mm of bone loss at 6 months follow-up period which was within the acceptable limits. The clinical parameters used in the present study such as plaque index, gingival index, sulcus bleeding index, to assess the peri-implant tissues, showed no significant effect on the crestal bone loss around implants. Also, there was an increase in the bone loss despite a decrease in the peri-implant probing depths, which can be attributed to the surgical trauma or increased occlusal load. However, it is necessary to have a large sample size and further longitudinal studies are required to evaluate the relationship between peri-implant soft and hard tissues in respect to the placement of implants.

### What is known about this topic

- It is important to remember that implants only replicate natural teeth and that the implant-mucosa-bone interface only approximates the natural periodontium;
- Lack of cementum and periodontal ligament, less vasculature and fibroblasts, a parallel orientation of supracrestal connective tissue;
- The subgingival location of crowns make the implant structures more susceptible to the development of inflammation and bone loss when exposed to plaque accumulation or microbial invasion.

### What this study adds

- Within the limitations of the present study, it was observed that following loading of implant, remodeling of the bone takes place, which is manifested as diminished vertical dimensions;
- Further longitudinal studies with a greater sample size are thus required to assess the bone loss and the effect of the soft tissue health on the bone levels around implants;
- Also, the type of abutment, implant length and diameter may influence the bone loss around implants so further evaluation should be done considering different abutment types, and different types of implants.

## References

[1] Thumati P, Padmaja S, Saritha H (2013). An evaluation of topographic changes in peri-implant hard and soft tissues using a standardized technique. J Dental Implants.

[2] Rajpal J, Gupta KK, Tandon P, Srivastava A, Chandra C (2014). Asssessment of hard and soft tissue changes around implants: a clinic-radiographic in vivo study. J Dental Implants.

[3] Anand U, Mehta DS (2012). Evaluation of immediately loaded dental implants bioactivated with platelet-rich plasma placed in the mandibular posterior region: a clinic-radiographic study. J Indian Soc Periodontol.

[4] Joly JC, de Lima AFM, da Silva RC (2003). Clinical and radiographic evaluation of soft and hard tissue changes around implants: a pilot study. J Periodontol.

[5] Patil RC, den Hartog L, van Heereveld C, Jagdale A, Dilbaghi A, Cune MS (2014). Comparison of two different abutment designs on marginal bone loss and soft tissue development. Int J Oral Maxillofac Implants.

[6] Lachmann S, Kimmerle-Muller E, Axmann D, Gomez-Roman G, Weber H, Haas R (2007). Reliability of findings around healthy implants in association with oral hygiene measures: a clinical, microbiological and immunological follow-up in edentulous patients. Clin Oral Implants Res.

[7] Weber HP, Crohin CC, Fiorellini JP (2000). A 5-year prospective clinical and radiographic study of no-submerged dental implants. Clin Oral Implants Res.

[8] Cardaropoli G, Lekholm U, Wennstrom JL (2006). Tissue alterations at implant-supported single-tooth replacements: a 1-year prospective clinical study. Clin Oral Implants Res.

[9] Nemcovsky CE, Artzi Z, Moses O, Gelernter I (2002). Healing of marginal defects at implants placed in fresh extraction sockets or after 4-6 weeks of healing: a comparative study. Clin Oral Implants Res.

[10] Mombelli A, Lang NP (1994). Clinical parameters for the evaluation of dental implants. Periodontol 2000.

[11] Lindquist LW, Rocker B, Carlsson GE (1988). Bone resorption around fixtures in edentulous patients treated with mandibular fixed tissue-integrated prostheses. J Prosthet Dent.

[12] DeAngelo SJ, Kumar PS, Beck FM, Tatkis DN, Leblebicioglu B (2007). Early soft tissue healing around one stage dental implants: clinical and microbiological parameters. J Periodontol.

[13] Ladwein C, Schmelzeisen R (2015). Nelson K, Fluegge TV, Fretwurst T. Is the presence of keratinized mucosa associated with periimplant tissue health: a clinical cross-sectional analysis. Int J Implant Dent.

[14] Klokkevold PR, Cochran DL (2009). Clinical aspects and evaluation of the implant patient; Carranza´a Clinical Periodontology. Sauders Elswvier.

[15] Chung DM, Oh TJ, Shotwell JL, Misch CE, Wang Hl (2006). Significance of keratinized mucosa in maintenance of dental implants with different surfaces. J Periodontol.

[16] Warrer K, Buser D, Lang NP, Karring T (1995). Plaque induced peri-implantitis in the presence or absence of keratinized mucosa. An experimental study in monkeys. Clin Oral Implants Res.

[17] Adibrad M, Shahabuei M, Sahabi M (2009). Significance of the width of keratinized mucosa on the health status of the supporting tissue around implants supporting overdentures. J Oral Implantol.

[18] de Rouck T, Collys K, Wyn I, Cosyn J (2009). Instant provisionalisation of immediate single-tooth implants is essential to optimizeesthetic treatment outcome. Clin Oral Implants Res.

[19] Albrektsson TO, Johansson CB, Sennerby L (1994). Biological aspects of implant dentistry: Osseointegration. Periodontol.

[20] Chou CT, Morris HF,Ochi S, Walker L, DesRosiers D (2004). AICRG, Part II: crestal bone loss associated with the Ankylos Implant: loading to 36 months. J Oral Implantol.

[21] Kan JYK, Runcharassaeng K, Umezu K, Kois JC (2003). Dimensions of peri-implant mucosa: an evaluation of maxillary anterior single implants in humans. J Periodontol.

[22] Chang M, Wennström JL (2010). Peri-implant soft tissue and bone crest alterations at fixed dental prostheses: a 3 year prospective study. Clin Oral Implants Res.

[23] Romeo E, Ghisolfi M, Rozza R, Chiapasco M, Lops D (2006). Short (8mm) dental implants in the rehabilitation of partial and complete edentulism: a 3 to 14-year longitudinal study. Int J Prosthodont.

[24] Cecchinato D, Bengazi F, Blasi G, Botticelli D, Cardarelli I, Gualini F (2008). Bone level alterations at implants placed in the posterior segments of the dentition: outcomes of submerged/ non submerged healing, A 5-year multicenter, randomized controlled clinical trial. Clin Oral Implants Res.

